# The roles of long noncoding RNA in epigenetic regulation

**DOI:** 10.1186/1756-8935-6-S1-O31

**Published:** 2013-03-18

**Authors:** Jeannie T Lee

**Affiliations:** 1 Howard Hughes Medical Institute, USA; 2 Department of Molecular Biology, Massachusetts General Hospital, USA; 3 Department of Genetics, Harvard Medical School, Boston, MA 02114, USA

## 

The X-linked region now known as the 'X-inactivation center' (Xic) was once dominated by protein-coding genes but, with the rise of Eutherian mammals some 150-200 million years ago, became infiltrated by genes that produce long noncoding RNA (lncRNA). Some of the noncoding genes have been shown to play crucial roles during X-chromosome inactivation (XCI), including the targeting of chromatin modifiers to the X. The rapid establishment of lncRNA hints at a possible preference for long transcripts in some aspects of epigenetic regulation. We will consider advantages lncRNA offers in delivering allelic, cis-limited, and locus-specific control. Unlike proteins and small RNAs, long ncRNAs are tethered to the site of transcription, and can therefore tag the allele of origin and mark unique regions in a complex genome. We will discuss general roles of lncRNA, with special consideration of XCI and developmental consequences when Xist RNA is aberrantly expressed. Like small RNAs, lncRNAs may emerge as powerful regulators of the epigenome.

